# Altered Cortical Responsiveness to Pain Stimuli after High Frequency Electrical Stimulation of the Skin in Patients with Persistent Pain after Inguinal Hernia Repair

**DOI:** 10.1371/journal.pone.0082701

**Published:** 2013-12-23

**Authors:** Emanuel N. van den Broeke, Lonneke Koeslag, Laura J. Arendsen, Simon W. Nienhuijs, Camiel Rosman, Clementina M. van Rijn, Oliver H. G. Wilder-Smith, Harry van Goor

**Affiliations:** 1 Department of Anesthesiology, Pain & Palliative Medicine, Radboud University Nijmegen Medical Centre, Nijmegen, The Netherlands; 2 Department of Surgery, Radboud University Nijmegen Medical Centre, Nijmegen, The Netherlands; 3 Department of Surgery, Catherina Hospital, Eindhoven, The Netherlands; 4 Department of Surgery, Canisius-Wilhelmina Hospital, Nijmegen, The Netherlands; 5 Donders Institute for Brain, Cognition and Behavior, Radboud University Nijmegen, Nijmegen, The Netherlands; University of Modena and Reggio Emilia, Italy

## Abstract

**Background:**

High Frequency electrical Stimulation (HFS) of the skin induces enhanced brain responsiveness expressed as enhanced Event-Related Potential (ERP) N1 amplitude to stimuli applied to the surrounding unconditioned skin in healthy volunteers. The aim of the present study was to investigate whether this enhanced ERP N1 amplitude could be a potential marker for altered cortical sensory processing in patients with persistent pain after surgery.

**Materials and Methods:**

Nineteen male patients; 9 with and 10 without persistent pain after inguinal hernia repair received HFS. Before, directly after and thirty minutes after HFS evoked potentials and the subjective pain intensity were measured in response to electric pain stimuli applied to the surrounding unconditioned skin.

**Results:**

The results show that, thirty minutes after HFS, the ERP N1 amplitude observed at the conditioned arm was statistically significantly larger than the amplitude at the control arm across all patients. No statistically significant differences were observed regarding ERP N1 amplitude between patients with and without persistent pain. However, thirty minutes after HFS we did observe statistically significant differences of P2 amplitude at the conditioned arm between the two groups. The P2 amplitude decreased in comparison to baseline in the group of patients with pain.

**Conclusion:**

The ERP N1 effect, induced after HFS, was not different between patients with vs. without persistent pain. The decreasing P2 amplitude was not observed in the patients without pain and also not in the previous healthy volunteer study and thus might be a marker for altered cortical sensory processing in patients with persistent pain after surgery.

## Introduction

High Frequency electrical Stimulation (HFS) of peptidergic primary C-fiber afferents induces Long-Term Potentiation (LTP) of excitatory synaptic transmission between these C-fibers and secondary lamina I dorsal horn neurons projecting to the parabrachial area in the brainstem [1]–[3]. As a consequence, these neurons show enhanced responsiveness to normal afferent input.

In healthy human volunteers, similar HFS of peptidergic C-fibers in human skin results in increased pain sensitivity to single electrical pain stimuli activating the conditioned pathway (i.e. homotopic effect)[4]–[7]. However, the effect of HFS is not restricted to the site of conditioning stimulation. After HFS, the non-conditioned skin surrounding the conditioned area also shows increased pain sensitivity, in particular to mechanical stimuli (i.e. heterotopic effect) [4], [7]–[10].

In order to investigate central nervous system responsiveness after HFS we measured Event-Related Potentials (ERPs) in response to painful electrical stimuli applied to the unconditioned surrounding skin [9]. ERPs are voltage polarity changes in the Electro-EncephaloGram (EEG), time-locked to the onset of a stimulus [11]. The EEG directly measures neuronal activity and the ERPs represent the synchronized activity of the underlying neural population. With the measurement of ERPs it is possible to study sequential stimulus processing of different brain structures in time [11]. By measuring ERPs before, directly after and thirty minutes after HFS we have observed an enhanced ERP amplitude around 100 ms (N1) thirty minutes after HFS at the conditioned arm compared to control arm in healthy human volunteers [9].

The fact that both phenomena; 1) a behaviorally increased sensitivity to mechanical stimulation and 2) an enhanced ERP N1 amplitude to electrical stimulation are observed at the same time and in the same skin area might suggest a relationship between the two. However, there was no significant correlation between the HFS-induced changes of mechanical punctate sensitivity and HFS-induced changes of evoked N1 amplitude (*r* = .35, *p* = .15) (unpublished observation, ref. 11) which might indicate that although both effects are induced by the same conditioning stimulation they probably reflect different underlying processes.

One possible interpretation of the enhanced ERP N1 amplitude after HFS could be that it is a reflection of enhanced saliency. Recently, Legrain et al. [12] proposed that the cortical network activated after painful stimulation represents, at least in part, a saliency detection system that is involved in detecting and orienting attention towards salient sensory events and reacting to the occurrence these events. The function of this cortical network is to facilitate the processing of behaviorally significant (e.g. potentially threatening) sensory input and to help select an appropriate response [12].

The aim of the present study was to investigate whether this ERP N1 effect induced after HFS could be a potential marker for altered cortical sensory processing in patients with persistent pain after surgery. Therefore the same experimental HFS paradigm as previously published [9] was used in patients with and without persistent pain after inguinal hernia repair. Our hypothesis that the effect of HFS on the ERP N1 amplitude thirty minutes after HFS is different in patients with pain compared to patients without pain.

## Materials and Methods

### Ethical Statement

Approval for the study was obtained from the medical and ethical review board committee region Arnhem-Nijmegen, Nijmegen, The Netherlands (NL 32573.091.10). All patients signed an informed consent form.

### Patients

Nineteen male patients (9 with and 10 without persistent pain) who underwent inguinal hernia repair (i.e. open anterior mesh-based repair procedure) 6–7 years ago were randomly recruited from a clinical trial database of the Canisius-Wilhelmina Hospital Nijmegen [13]. Patients (with and without pain) were excluded from the study if they: (i) had a psychiatric or neurological condition (neurological symptoms as a result of the inguinal hernia repair were allowed), (ii) used pain medication or other medication that potentially affects brain processing like anti-depressants, anti-psychotics, anti-convulsants, benzodiazepines and narcotics, (iii) suffered from any pre-existing pain (i.e. before surgical intervention) or pain syndrome, (vi) used recreational drugs and (v) had any sign of tissue damage at – or near – the site of experimental stimulation (vi) participated in other research, (vii) had a (repaired) recurrent hernia. The patients in the control group (without pain group) were excluded if they reported any type of pain.

### Demographic and Clinical Characteristics

The definition of the two inguinal hernia repair groups (with and without pain) was based on a question (obtained via interview by telephone) asking whether the patient experienced ongoing pain (yes or no) as a result of the inguinal hernia repair treatment. For confirmation, the same question was asked again on the day of measurement, together with an additional question (only if the patient experienced pain) regarding pain intensity as a measure of past experienced pain load (*‘What is the averaged intensity of the inguinal hernia repair-related pain during the last three months on a numeric 0–10 rating scale?’)*. Other demographic and clinical characteristics that were obtained are: *age*, body weight, length, operation type, medication use and co-morbidity (see table 1).

**Table 1:** Demographic and clinical characteristics of the patients with pain (N = 8) and without pain (N = 7).

*Patient*	*Age* *(years)*	*Weight* *(kg)*	*Length* *(m)*	*Surgical* *intervention*	*Persistent* *pain?*	*Pain intensity* *averaged* *over last* *three months*	*Pain intensity* *at day of* *measurement*	*Location* *of the pain*	*Co-morbidity*
1	37	73	1.75	Mesh	yes	–	6	right	
2	42	82	2.02	Mesh	yes	7	0	Right	Low back pain
3	65	90	1.75	Lichtenstein	yes	3	3	Left	Painful shoulder; Diabetis mellitus II
4	46	118	1.98	Lichtenstein	yes	8	3	Right	Painful wrists over whole body
5	56	79	1.83	Lichtenstein	yes	3.5	0	left	
6	48	86	1.72	Mesh	yes	3	0	Left	Pain in right shoulder
7	58	80	1.86	Mesh	yes	2	0	left	
8	70	62	1.72	Mesh	yes	7	0	right	
***Mean (SD)***	53 (11)	84 (16)	1.83 (0.12)			4.8 (2.4)	1.5 (2.3)		
1	58	70	1.73	Lichtenstein	no				Astma
2	58	74	1.82	Prolene	no				
3	62	75	1.60	Lichtenstein	no				Hypertension
4	59	72	1.90	Mesh	no				
5	62	72	1.68	Lichtenstein	no				
6	46	95	1.81	Mesh	no				
7	68	72	1.60	Prolene	no				Hypertension; Diabetis mellitus II
***Mean (SD)***	59 (7)	76 (9)	1.73 (0.12)						

Data about the type of pain and pain-related sensory signs in the patients with pain were collected using the DN4 (Douleur Neuropathique 4) questionnaire [14], [15] (see table 2). This questionnaire includes pain descriptors as well as three clinical tests reflecting altered somatosensory processing. For measuring hypoesthesia to touch a Senselab brush-05 (Somedic) was applied on different skin sites in the location of the pain. For measuring hypoesthesia to pinprick a Semmes-Weinstein monofilament (nr. 5.07, 10.0 g) was applied to different skin areas in the location of the pain. For measuring brush evoked or increased pain within the location of pain, the same brush as for hypoesthesia was used. The effects of stimulation of the first two clinical tests (hypoesthesia to touch and pinprick) were quantified by comparing the skin sites in the location of pain to a control site on the contralateral body site. It is important to mention that in this study the DN4 questionnaire is not used as a screening or diagnostic instrument for neuropathic pain because at present it is not validated for this purpose in this population of surgical patients. Thus we used the DN4 exclusively to collect data regarding the clinical qualitative characteristics of the pain syndrome.

**Table 2:** Results of the DN4 questionnaire.

	Pain Charcteristics			Symptoms associated with the pain				Symptoms present in pain location		
	*Burning*	*Painful* *Cold*	*Electrical* *shocks*	*Tingling*	*Pins and* *needles*	*Numbness*	*Itching*	*Hypoesthesia* *to touch*	*Hypoesthesia* *to pinprick*	*pain after* *Brushing*
1	**−**	**−**	**−**	**−**	**−**	**−**	X	**−**	X	**−**
2	**−**	**−**	**−**	X	X	**−**	**−**	X	**−**	**−**
3	X	**−**	X	X	X	**−**	**−**	**−**	**−**	X
4	X	**−**	**−**	X	X	X	X	X	X	**−**
5	**−**	**−**	**−**	**−**	X	**−**	**−**	**−**	**−**	**−**
6	**−**	**−**	**−**	**−**	X	**−**	**−**	**−**	**−**	**−**
7	X	X	**−**	**−**	**−**	X	X	X	**−**	**−**
8	**−**	**−**	**−**	**−**	**−**	**−**	**−**	X	X	X
% patients	37.5%	12.5%	12.5%	37.5%	62.5%	25.0%	37.5%	50.0%	37.5%	22.0%

Shown are the individual patient characteristics as well as group percentages regarding type of pain, associated symptoms and clinical tests. **−** = no, X = yes.

### Design

#### Experimental conditioning: high frequency electrical stimulation (HFS)

All patients received trains of 100 Hz (pulse width; 2 ms) for 1 sec. repeated 5 times at 10 sec intervals with an intensity of 20 × detection threshold on the forearm 5 cm distal to the cubital fossa. The stimulation trains were delivered via a constant current stimulator (Digitimer DS7A, Digitimer UK) and a specifically designed electrode able to activate peptidergic nociceptive afferents in the skin [4]. The electrode consists of 16 blunt stainless steel pins with a diameter of 0.2 mm protruding 1 mm from the base. The 16 pins are placed in a circle with a diameter of 10 mm and serve as cathode. A stainless steel reference electrode which serves as anode is concentrically located and has an inner diameter of 22 mm and an outer diameter of 40 mm. This electrode is specially designed to activate superficial nociceptive afferents with less concomitant recruitment of tactile afferents. Trains of high frequency stimulation delivered through this electrode are perceived as moderate to strongly painful and increase gradually after each train [9]. In order to avoid interference of lateral dominance, the stimulated arm (HFS) was balanced (dominant or not dominant) across patients. The opposite arm to the one receiving conditioning stimulation served as control.

### Behavioral Measurements

#### High frequency conditioning stimulation

Changes in pain perception during experimental conditioning stimulation (HFS) were tested by asking the patients after each train to rate the amount of pain on a Visual Analogue Scale (VAS) ranging from 0 cm = “no pain” to 10 cm = “unbearable pain”.

#### Test stimuli applied to the heterotopic skin area

In order to quantify changes in central nervous system responsiveness as a result of experimental conditioning stimulation, blocks of twenty single painful electrical pulses (monopolar square wave; duration 0.5 ms) were applied to both arms (conditioned and control) before (t0), directly after (t1) and thirty minutes (t3) after experimental conditioning stimulation. We chose thirty minutes as a late measurement after conditioning stimulation because Klein et al. [16] showed that punctate hyperalgesia develops immediately after HFS and then increases slightly over the next 40 min, peaking between 40 and 60 min after HFS. Thus we chose thirty minutes after HFS in order to be sure the effect was well-established without being in the declining phase.

For the conditioned arm, the stimuli were applied at 2.5 cm outside the area of conditioning stimulation; on the control arm the same area was stimulated. The pulses were delivered with a random inter-pair interval ranging from 7 to 10 seconds via a concentric electrode (CE). Stimulation with this electrode produces a well localized pinprick-like painful sensation [17].

In order to quantify the amount of pain as a result of this test stimulation, subjects were asked to rate four times within the block of twenty stimuli, i.e., at random times within a set of 5 single pulses, the pain intensity of the last received stimulus on a VAS. The VAS ranged from 0 cm = “no pain” to 10 cm = “unbearable pain” and was used by the subject by moving the mouse pointer (vertical line) on a horizontal bar.

The electrical test stimuli were delivered via a constant current stimulator (Digitimer DS7A, Digitimes, Hertfordshire, UK) and with an intensity of 150% of the individual pain threshold. This individual pain threshold was determined at the beginning of the experiment by delivering an ascending sequence of increased current intensities (single square wave current pulse; duration 5 ms) starting from 0 mA and with steps of 0.1 mA. This procedure stopped when the pain threshold (pricking painful sensation) was achieved, as verbally reported by the subjects. This threshold determination protocol was performed twice and the mean was used in the experiment.

During stimulation, subjects were comfortably seated in a chair and were instructed to passively perceive the stimuli with eyes closed, without making any movements. A computer display was placed in front of the subject (0.5 m) together with a computer mouse. The display was used to display the VAS, preceded by a tone (65 dB). Participants were instructed to open their eyes after the tone and use the mouse to mark the VAS, after which they closed their eyes again.

### Electrophysiological Measurements

In order to measure the evoked brain responses (ERPs) as a result of the heterotopic test stimulation, a multi-channel (28 channels) EEG (Brainvision system) was recorded (band-pass 0.1–100 Hz, sample frequency 500 Hz). The electrodes were mounted in an elastic electrode-cap and arranged according to the international 10–20 system. Eye movements were detected by horizontal and vertical Electro-OculoGram (EOG) recordings (channels TP9, P09, TP10, P010 from the cap were used for the EOG recordings). Horizontal EOG was measured from the outer canthus of the left eye, and vertical EOG supra orbitally to the left eye. Impedance was kept under the 20 kΩ for all leads. The (separate) reference electrode was placed in FCz.

### Procedure

At the beginning of the experiment individual pain thresholds for the electrical pinprick-like test stimulation were determined. The arm on which this pain threshold was determined (conditioned or control arm) was balanced across patients. After this pain threshold determination patients received two blocks (one at each arm) of electrical pain stimuli (t0). The sequence regarding which arm was tested first was balanced across patients. After the baseline measurement (t0) the experimental conditioning (HFS) followed. After receiving conditioning stimulation two post measurements t1 (directly after conditioning stimulation) and t2 (thirty minutes after conditioning stimulation) followed. The procedures for these two post measurements were the same as for the pre measurement. Patients were instructed not to consume caffeine-containing beverages for twelve hours before the start of the experiment to avoid the caffeine-induced theta decrease in EEG [18].

### Signal Analysis

**Figure 1:**
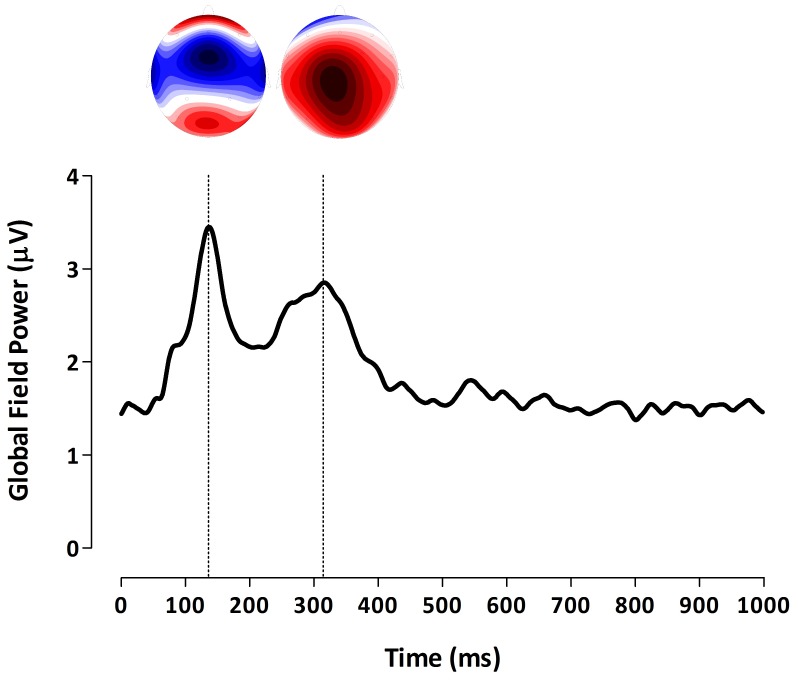
Grand average global field power (GFP) and corresponding topographic representations. **A)** Grand average GFP. The dotted lines indicate peak latency of the different ERP components. Two different components can be identified: (1) A negative voltage between 100–200 ms, maximal at Cz, labeled as N1; (2) A positive voltage between 240–380 ms, maximal at Cz, labeled as P2. **B)** Topographic representations of the ERP components at the ERP latencies. To best illustrate the maximal activity in each representation we adjusted the scale to its maximal absolute values (for increases and decreases in voltages). As a result the scale differs between the different representations and is therefore left out.

The EEG was analyzed offline using the software Brain Vision Analyzer v. 2.0 and Matlab 2011a. As a first step the continuous EEG was referenced to a common average (i.e. all electrodes). Next, the EEG signal (500 Hz) was high-pass filtered at 1 Hz and low-pass filtered at 30 Hz. Based on the onset of the stimulus, the EEG was segmented into epochs from −100 ms pre-stimulus to 1000 ms post-stimulus with a total period of 1100 ms. Bad segments containing ocular artifacts were corrected using the Gratton-Coles method [19]. Segments were also inspected for other artifacts like muscle or jaw and line noise activity and were removed if necessary. As a last step baseline correction (−100–0 ms) was applied to all segments. For each patient separately, all segments were averaged to obtain an averaged subject-specific event-related potential waveform. ERP components were defined in terms of their latency and topographic distribution. For this the grand average global field power (GFP) of all patients was calculated [20], [21]. Subsequently, we calculated the topographic voltage distribution corresponding to the ERP latencies identified in the GFP plot. Then we identified the electrode in the topographic plot which shows the maximal activity and used this electrode for subsequent analysis. To insure accurate identification of point of maximal activity we also inspected the grand average ERPs (of all electrodes) for all patients. Based on the grand average GFP and corresponding topographic representations of all patients (N = 19) shown in figure 1, we defined two distinctive ERP components: 1) A negative voltage between 100–200 milliseconds (ms), maximal at electrode Cz, which we label as N1 and 2) A positive voltage between 240–380 ms, maximal at Cz, which we label as P2.

Individual ERP latencies were determined in the individual GFP plot corresponding to the windows of the grand average GFP latencies [20]. The mean amplitude of each ERP component was calculated at the individual GFP-latency ±5 ms at the electrode of maximal activity [20]. The rationale for using the mean activity instead of the more commonly used maximal peak value (baseline-to-peak) is that the fewer trials included in the subject-specific average, the more residual noise is superimposed on the maximal peak, and thus the more the maximal peak of the subject-specific average will be determined by residual noise rather than by the peak of interest. Therefore we calculated the mean amplitude instead of the maximal peak amplitude because the former value is more stable and representative of evoked activity [22].

### Statistical Analysis

For statistical analysis the software SPSS v. 18.0 was used (SPSS Inc., Chicago, IL, USA). To statistically test whether there are differences in behavioral and electrophysiological measures after HFS between the two groups a General Linear Model (GLM) mixed design ANOVA analysis was performed. The within-subject factors were *TIME of measurement* (pre (t0) vs. post (t1, t2)) and *ARM* (control vs. conditioned). The between-subject factor was *GROUP* (patients with vs. without pain). The dependent variables were the pain VAS-score observed after each HFS train and during test stimulation and ERP (N1 and P2) amplitude and latency. In those cases where the data violated the assumption of sphericity the F-value was corrected by Greenhouse-Geisser. In all tests the level of significance was set at *p*<.05 (two-sided). For post hoc testing the *p*-value was Bonferroni corrected for the number of tests.

## Results

In four patients (three in the without pain group and one in the pain group) the conditioning stimulation had to be stopped before receiving all five trains because they could not tolerate the stimulation anymore. The patient in the pain group received only one train, one patient of the group without pain received two trains and two patients received three trains. They were excluded in the further statistical analyses.

### Demographic and Clinical Characteristics

The demographical and clinical characteristics of both patient groups are shown in table 1. Independent t-tests revealed no statistically significant differences in age, length or weight between the two groups of patients. Table 2 shows the clinical pain characteristics as observed in the patients with persistent pain.

### Electrical Detection Thresholds for Conditioning Stimulation

The mean (and standard deviation) electrical detection thresholds used for conditioning stimulation (HFS) were 5.14 (1.68) mA for the patients without pain and 4.88 (1.96) mA for the patients with pain. No statistically significant differences in detection thresholds between the two groups were observed (independent t-test: *p*>.7).

### Stimulation Intensities for the Electrical Test Stimuli

The mean (and standard deviation) intensities used for the electrical test stimuli were 3.30 (1.35) mA for the patients without pain and 3.51 (1.43) mA for the patients with pain. No statistically significant differences in electrical stimulation intensities between the two groups were observed (independent t-test: *p*>.7).

### Behavioral Measurements

#### Pain intensity rating after each train of HFS

Mean (and standard deviations) pain ratings for each subsequent train are: 5.8 (2.1), 6.3 (1.8), 6.8 (1.7), 7.0 (1.8), 6.7 (2.4) for the patients without pain and 5.3 (2.5), 6.1 (2.5), 6.7 (2.1), 7.1 (2.0), 7.2 (2.2) for the patients with pain. The GLM mixed design ANOVA analysis revealed a significant main effect of *Time* (F_Greenhouse-Geisser_ (1.520,19.759) = 10.607, *p* = .002, η^2^ = .449). This means that the pain VAS-score differed between the different trains across all patients (with and without pain). Univariate within-subject contrasts showed that the perceived pain intensity statistically increased:

between train 1 (M = 5.5) and train 2 (M = 6.2) : F (1,13) = 16.933, *p* = .001, η^2^ = .566, and
between train 2 and train 3 (M = 6.8) : F (1,13) = 11.060, *p* = .005, η^2^ = .460, but *not* between:
train 3 and train 4 (M = 7.1) : F (1,13) = 3.625, *p* = .079, η^2^ = .218, and
train 4 and train 5 (M = 7.0) : F (1,13) = .322, *p* = .580, η^2^ = .024.

There were no statistically significant differences in perceived pain intensity (VAS scores) obtained after each train between the two groups (see table 3).

**Table 3:** Mean (and SD) pain VAS-scores, ERP amplitude and latencies of the patients without pain (N = 7) and patients with pain (N = 8).

			T0		T1		T2	
			*Control* *arm*	*Conditioned* *arm*	*Control* *arm*	*Conditioned* *arm*	*Control* *arm*	*Conditioned* *arm*
**ERP N1**	Amplitude (µV)	Pain	−4.0 (4.3)	−0.3 (4.8)	−3.2 (3.7)	−1.9 (4.5)	−1.1 (3.6)	−4.9 (4.5)
		Without pain	−3.2 (3.0)	−2.7 (3.1)	−1.6 (2.0)	−1.7 (3.5)	−1.7 (1.9)	−2.9 (3.0)
	Latency (ms)	Pain	151.5 (18.7)	165.8 (31.7)	146.3 (14.7)	153.3 (30.2)	142.3 (32.9)	137.0 (27.6)
		Without pain	136.9 (19.4)	142.3 (20.6)	143.7 (11.9)	132.9 (22.5)	136.6 (11.1)	134.3 (15.2)
**ERP P2**	Amplitude (µV)	Pain	3.9 (2.4)	4.3 (1.6)	3.5 (2.4)	3.6 (1.7)	5.1 (2.1)	2.1 (1.8)
		Without pain	4.2 (1.7)	4.5 (2.5)	4.3 (1.7)	6.1 (2.1)	5.0 (1.1)	6.2 (2.3)
	Latency (ms)	Pain	318.0 (41.0)	321.5 (23.3)	308.8 (41.9)	311.3 (41.2)	305.5 (32.4)	318.3 (41.6)
		Without pain	301.4 (30.5)	280.0 (35.1)	281.7 (28.2)	289.1 (27.2)	289.1 (39.0)	286.3 (37.8)
**Pain intensity**	VAS (0–10 cm)	Pain	2.7 (1.6)	3.1 (1.9)	2.4 (2.1)	1.9 (1.7)	2.4 (1.8)	2.2 (2.1)
**test stim**		Without pain	3.1 (1.5)	3.1 (1.7)	2.6 (1.9)	2.7 (1.9)	2.5 (1.5)	2.8 (2.0)

#### Perceived pain intensity during test stimulation

The GLM mixed design ANOVA analysis revealed a marginally significant main effect of *Time* (F_Greenhouse-Geisser_ (1.079, 14.022) = 4.363, *p* = .053, η^2^ = .251). This means that when we ignore the different arms (control vs. conditioned) the pain VAS-score differed between the different measurements across all patients (with and without pain). Univariate within-subject contrasts showed that the perceived pain intensity (VAS-scores) was marginally signifcantly different between t0 (M = 3.0) and t1 (M = 2.4) [F (1, 13) = 4.394, *p* = .056, η^2^ = .253] and also between t0 (M = 3.0) and t2 (M = 2.5) [F (1, 13) = 4.640, *p* = .051, η^2^ = .263]. There were no statistically significant differences in perceived pain intensity between the two groups.

### Electrophysiological Measurements

**Figure 2:**
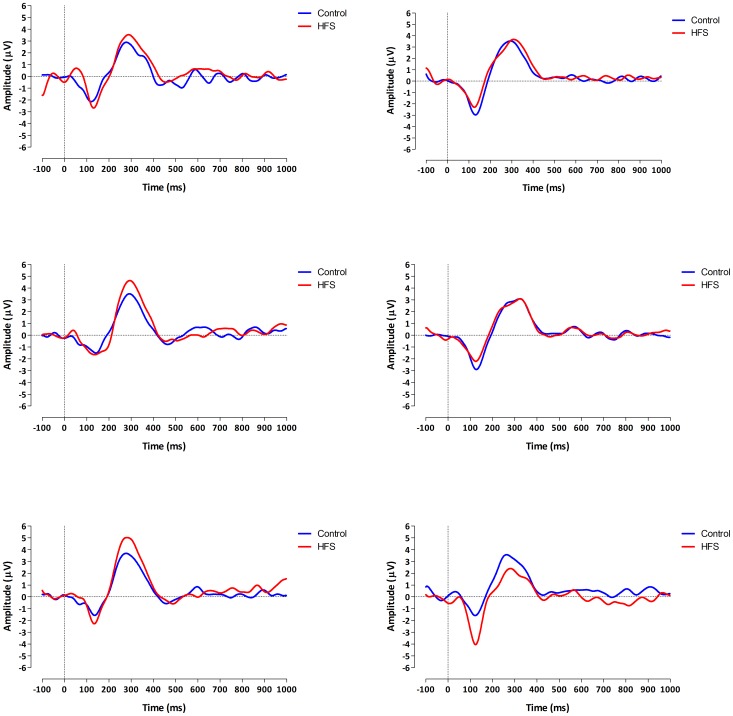
ERP waveforms. Grand average ERPs observed from Cz for both the conditioned and control arm. Left column are the ERPs of the patients without pain (N = 7) and right column are the ERPs of the patients with pain (N = 8). Upper row is t0 (before HFS), middle row is t1 (directly after HFS) and lowest row is t2 (thirty minutes after HFS). Upward deflection is positive charge and downward is negative charge. Representations of ERPs are with respect to common reference.

The grand average evoked potential waveforms for both groups (with and without pain) and each measurement (t0, t1 and t2) and arm (conditioned vs. control) are shown in figure 2. The mean (and SD) N1 and P2 amplitudes and latencies are summarized in table 3.

#### N1 amplitude

**Figure 3:**
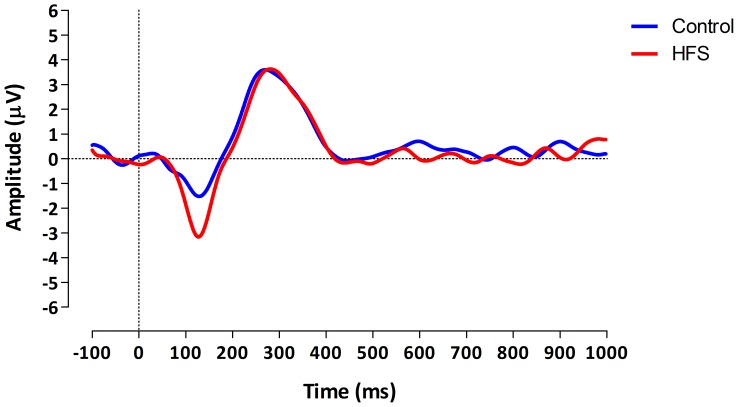
ERP N1 amplitude difference between conditioned and control arm thirty minutes after HFS across all patients.

Regarding primary outcome, the GLM mixed design ANOVA analysis revealed a statistically significant *Time × Arm* interaction effect for the N1 amplitude (F (2,26) = 5.435, *p* = .011, η^2^ = .295). This means that the N1 amplitude was statistically significantly different between the two arms at the different measurements across all patients (with and without pain). The univariate within-subject contrasts revealed that the N1 amplitude was different between the two arms thirty minutes after experimental conditioning stimulation (F (1, 13) = 8.329, *p* = .013, η^2^ = .391). The N1 amplitude observed at the conditioned arm (M = −4.0) was larger than the N1 amplitude observed at the control arm (M = −1.4) (fig. 3).

Post-hoc testing revealed that thirty minutes after HFS the N1 amplitude of the control arm (M = −1.4) was significantly lower than the amplitude observed at baseline (pre HFS, M = −3.6) [paired t-test; t (14) = −2.737, *p* = .016]. There were no statistically significant differences in N1 amplitudes between the two groups.

#### N1 latency

No statistically significant differences on N1 latency were observed after conditioning stimulation between arms and/or groups.

#### P2 amplitude

For the P2 amplitude the GLM mixed design ANOVA analysis revealed a statistically significant *Time × Arm* interaction effect (F (2,26) = 3.676, *p* = .039, η^2^ = .220). This means that the P2 amplitude statistically differs between the two arms at the different measurements across all patients (with and without pain). No univariate within-subject contrasts were statistically significant.

The GLM mixed design ANOVA analysis also revealed a significant *Time x Group* interaction effect (F (2, 26) = 3.868, *p* = .034, η^2^ = .229). This means that when we ignore the different arms (conditioned vs. control) the P2 amplitude differed between the two groups at the different measurements.

The univariate within-subject contrasts revealed that the amplitude was statistically significant different between the two groups between t0 (M_pain_ = 4.1, M_without pain_ = 4.4) and t2 (M_pain_ = 3.6, M_without pain_ = 5.6) [F (1, 13) = 8.317, *p* = .013, η^2^ = .390].

The GLM mixed design ANOVA also revealed a statistically significant *Time × Arm x Group* interaction effect (F (2, 26) = 5.108, *p* = .013, η^2^ = .282). The P2 amplitude was statistically significantly different between the two arms at the different measurements and between the two groups. Only the univariate contrast that compared the P2 amplitude between t0 and t2 regarding two arms (conditioned vs. control arm) and between the two groups (patients with vs. without pain) was significant (F (1, 13) = 6.488, *p* = .024, η^2^ = .333). At baseline the difference in P2 amplitude between the two arms was similar for both groups: patients without pain (M_control arm = _4.2, M_conditioned arm = _4.5); patients with pain (M_control arm = _3.9, M_conditioned arm = _4.3). Thirty minutes after HFS (i.e. t2) this difference became larger in the patients with pain compared to the patients without pain. The P2 amplitude of the control arm in both groups was similar at t2: patients without pain (M_control arm = _5.1); patients with pain (M_control arm = _5.0), while the P2 amplitude of the conditioned arm in the patients with pain was smaller (M_conditioned arm = _2.1) than in the patients without pain (M_conditioned arm = _6.2).

Post hoc testing revealed that in the patients with pain the P2 amplitude observed at the conditioned arm at t2 (M = 2.1) significantly decreased in comparison to t0 (M = 4.3) [t (7) = 3.636, *p*<.012].

#### P2 latency

No statistically significant differences on P2 latency were observed after conditioning stimulation between arms and/or groups.

## Discussion

The aim of this study was to test the hypothesis that the effect of HFS on the ERP N1 amplitude thirty minutes after HFS is different in patients with pain compared to patients without pain. In agreement with the previous healthy volunteer study [9] we observed an enhanced N1 amplitude at the conditioned arm compared to control arm thirty minutes after HFS across all patients (with and without pain). More importantly, we did not observe statistically significant differences in N1 amplitudes between the two groups of patients.

Surprisingly, we did observe statistically significant ERP P2 amplitude differences thirty minutes after HFS between the two groups of patients. Post hoc testing revealed a significant decrease of the P2 amplitude at the conditioned arm in the patients with pain. This P2 amplitude effect might be a marker for altered cortical sensory processing in patients with persistent pain.

### Heterotopic Effects after HFS

High frequency electrical stimulation of human skin typically induces a behaviorally increased sensitivity to mechanical stimulation in the surrounding unconditioned skin [4], [7]–[10]. Surprisingly, this behavioral effect is not observed for electrical stimulation as has been observed previously [8], [9] and in the present study. One explanation could be that the induced increased sensitivity after HFS mainly involves mechanosensitive afferents and can only be detected with mechanical stimulation. An electrical stimulus activates multiple types of afferent fibers and its percept is thus a mixture of percepts which could have blurred the perceptual effect of increased mechano-specific sensitivity. An alternative explanation could be that HFS induces, simultaneously with the increased sensitivity at the mechano-specific fibers, an opposite and possibly more pronounced effect (analgesia or hypoesthesia) at other fibers which are not mechano-specific.

In contrast to the lack of increase of perceived pain intensity after HFS, we did observe a statistically significant decrease directly, and thirty minutes after HFS. One explanation could be that this decrease reflects habituation; a decrease in response to a stimulus when that stimulus is presented repeatedly [23]. Alternatively, the observed VAS effect could be similar to the effect observed in heterotopic noxious conditioning stimulation (HNCS) paradigms [24]. In these paradigms, before and after a ‘conditioning’ stimulus (e.g. Ice water bath) the response (for example perceived pain intensity) to a heterotopically applied ‘test’ stimulus is measured. It has been observed that the perceived pain intensity to the ‘test’ stimulus after the conditioning stimulus is reduced in comparison to before and this is believed to be a manifestation of diffuse noxious inhibitory controls (DNIC) [24], [25], suggesting the involvement of the descending neural endogenous analgesia system [24], [25].

Despite the lack of sensitivity of an electrical stimulus in detecting the behavioral correlate of the induced heterotopically increased sensitivity after HFS we continued to use it in the present experiment. Firstly, because we wanted to compare the outcomes of this experiment with the ones of the healthy volunteer study [9]. Secondly, and more practically, we did not have access to a mechanical pinprick system that is connected to an EEG system. The implementation of such a technique is quite challenging because one has to make sure that the evoked EEG is exactly time locked to the onset of the stimulus.

### The Evoked ERP N1 Effect after HFS

In agreement with the healthy volunteer study [9] we showed that the ERP N1 amplitude, evoked by electrical stimulation, was significantly enhanced at the conditioned arm thirty minutes after HFS in comparison to control arm. Interestingly, Iannetti et al. [26] recently measured ERPs in response to mechanical pinprick stimulation applied to the heterotopic skin area after capsaicin in healthy volunteers. The authors observed a similar ERP effect after capsaicin as we did after HFS. Also Maihöfner et al. [27] observed similar results by using MEG and an electrical pain-inducing conditioning protocol. After painful electrical conditioning stimulation the authors observed enhanced event-related field activity, present around 100 milliseconds, in response to mechanical stimulation applied in the surrounding unconditioned skin. These findings indicate that the electrophysiological correlate, present around 100 milliseconds can be evoked independently of the used pain-inducing conditioning protocol and test stimulus (electrical vs. mechanical).

The present study shows that there are no differences in ERP N1 effect -induced after HFS- between the two groups of patients. Nevertheless, it would be interesting to investigate in detail what process this ERP N1 effect reflects.

### The Evoked ERP P2 Effect after HFS

Interestingly, thirty minutes after HFS a decrease of P2 amplitude at the conditioned arm is observed in the patients with pain. This effect is unexpected because it is not observed in the previous healthy volunteer study [9] and also not, as the present study shows, in the patients without pain. It would be interesting to know what kind of process(es) this ERP activity reflects, but at present this is still unknown.

The amplitude decrease might reflect a similar phenomenon as the one observed by Valeriani et al [28]. After capsaicin application they observed a reduction of the laser evoked potential evoked from the adjacent skin area (i.e. area of secondary hyperalgesia). By using a dipole source analysis the authors further demonstrated that this amplitude decrease probably involves activity originating from the cingulate cortex. The authors interpreted their amplitude reduction as increase cortical inhibition of cingulate activity triggered by the conditioning stimulus [28]. However, there are differences between the two studies. For example, in the study of Valeriani et al. they used capsaicin as conditioning stimulus, while we used electrical stimulation. Furthermore, their test stimuli were laser evoked potentials while in the present study electrical stimuli were used. Moreover, their study involves healthy volunteers, however, in our previous healthy volunteer study as well as in our patients without pain we did not observed such an amplitude reduction after HFS, which raises the following question; if the observed amplitude reduction in the present study reflects the same underlying process as the one underlying the amplitude reduction in the study of Valeriani et al., why does it only occur in the patients with pain?

### Methodological Considerations

#### Evoked potential waveform

Electrical stimulation applied via a concentric intra-epidermal electrode seems capable in activating Aδ fibers selectively provided that low stimulation intensities are used (i.e., 2x absolute detection threshold) [29]. However, Mouraux et al. also showed that this method loses its selectivity when higher stimulation intensities are used (i.e., above 2.5. mA). This is probably due to the fact that the electrical current penetrates deeper into the skin and thus also activates low threshold mechanoreceptors (tactile Aβ fibers) which have a lower activation threshold. As a consequence the simultaneously recorded evoked brain responses may reflect Aβ rather than Aδ fiber evoked responses [29]. In the present study we used relatively high stimulation intensities (Mean 3.30 (SD = 1.35) mA for the patients without pain and 3.51 (1.43) mA for the patients with pain), which are clearly above 2.5 mA. Therefore, we suggest that the ERPs of the present study likely reflect Aβ rather than Aδ evoked brain responses.

#### Sample size

One of the important methodological limitations of this study is the small sample size. Nevertheless, substantial effect sizes are observed which supports the relevance of our findings. Hence, future studies with larger sample sizes are necessary to confirm the results of the present paper.

## Conclusion

This is the first study that investigated the effects of HFS in patients with persistent pain after surgery. It shows that the ERP N1 effect induced after HFS was not different between patients with and without persistent pain. Surprisingly, we did observe a difference in P2 amplitude between the patients with and without pain. The decreased P2 amplitude might be a marker for altered cortical sensory processing in patients with persistent pain after surgery.
